# Humanized mice for investigating sustained *Plasmodium vivax* blood-stage infections and transmission

**DOI:** 10.1038/s41467-022-31864-6

**Published:** 2022-07-15

**Authors:** Camilla Luiza-Batista, Sabine Thiberge, Malika Serra-Hassoun, Flore Nardella, Aurélie Claës, Vanessa C. Nicolete, Pierre-Henri Commère, Liliana Mancio-Silva, Marcelo U. Ferreira, Artur Scherf, Sylvie Garcia

**Affiliations:** 1 Institut Pasteur, Université Paris Cité, Inserm U1201, CNRS EMR9195, Unité de Biologie des Interactions Hôte-Parasite, 75015 Paris, France; 2 Sorbonne Université, École Doctorale Complexité du Vivant, Paris, France; 3 Department of Parasitology, Institute of Biomedical Sciences, University of São Paulo, São Paulo, Brazil; 4 Institut Pasteur, Université Paris Cité, Cytometry and Biomarkers UTechS, CRT, 75015 Paris, France

**Keywords:** Parasite biology, Malaria

## Abstract

*Plasmodium vivax* is the most widespread human malaria parasite. Due to the presence of extravascular reservoirs and relapsing infections from dormant liver stages, *P. vivax* is particularly difficult to control and eliminate. Experimental research is hampered by the inability to maintain *P. vivax* cultures in vitro, due to its tropism for immature red blood cells (RBCs). Here, we describe a new humanized mice model that can support efficient human erythropoiesis and maintain long-lasting multiplication of inoculated cryopreserved *P. vivax* parasites and their sexual differentiation, including in bone marrow. Mature gametocytes were transmitted to *Anopheles* mosquitoes, which led to the formation of salivary gland sporozoites. Importantly, blood-stage *P. vivax* parasites were maintained after the secondary transfer of fresh or frozen infected bone marrow cells to naïve chimeras. This model provides a unique tool for investigating, in vivo, the biology of intraerythrocytic *P. vivax*.

## Abstract (web summary)

Plasmodium vivax is the most widespread human malaria parasite but due to its tropism for immature red blood cells in vitro culturing and studies are difficult. Here, LuizaBatista et al. generate a humanized mouse supporting human erythropoiesis and allowing asexual and sexual development of P. vivax in bone marrow and peripheral blood, as well as transmission to mosquitoes.

## Introduction

Malaria poses a major public health burden worldwide, with 241 million clinical cases and 627,000 deaths in 2020, according to the WHO^1^. Two *Plasmodium* species are responsible for the vast majority of human infections: *P. falciparum*, which predominates in Africa and causes severe disease, and *P. vivax*, the globally widespread parasite associated with debilitating illness, and occasionally, severe complications^2^. The elimination of malaria remains hampered by drug resistance and the lack of an efficient vaccine. In addition, the major challenges in eradication strategies include the dormant liver stages in *P. vivax* (called hypnozoites)^3^, the extravascular parasite reservoirs, such as bone marrow (BM)^4,5^, and the early circulation of  transmissive stages (gametocytes). Designing new control strategies requires a good understanding of *P. vivax* biology and host-parasite interactions; however, a major roadblock is the inability to maintain continuous cultures in vitro that can sustain the parasite erythrocytic cycle^6^. In part, this inability is due to the parasite’s tropism for CD71^+^ immature RBCs, which are rare in peripheral blood (PB)^7^. Humanized mice that can maintain *P. vivax* infections through de novo production of CD71^+^ human RBCs might circumvent this drawback. However, current models are limited by poor human erythropoiesis upon engraftment of hematopoietic stem and progenitor cells (HSPCs)^8^, which prevents *P. vivax* from mounting a blood-stage infection.

Here, we describe a new humanized mouse model, which exhibits human erythropoiesis upon HSPC transplantation. After injecting cryopreserved *P. vivax*-infected RBCs, these mice could support de novo *P. vivax* productive infections and *P. vivax* sexual differentiation. We show that the model reproduced several features of the natural infection. These features included parasite localization in the BM, with a prevalence of sexual-stage gametocyte and their later transmission to *Anopheles* mosquitoes. This process occurred even in low parasitemia settings and was followed by the production of salivary gland sporozoites. In addition, *P. vivax* could be maintained when BM cells from infected chimeras were transferred to secondary naive chimeras; this strategy provides a unique tool for maintaining *P*. *vivax* samples isolated from patients.

## Results

### HIS-HEry mice support human erythropoiesis

**Fig. 1:**
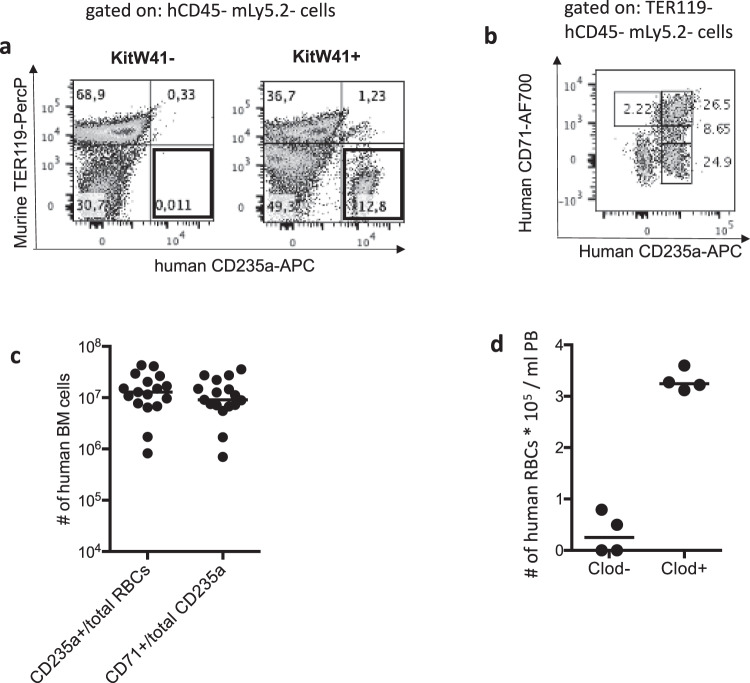
HIS-HEry chimeras support human erythropoiesis. BM cells from 20- to 24-week-old HIS-HEry (KitW41 + ) mice were analyzed with flow cytometry. **a** Representative plots show human CD235a + RBCs (highlighted quadrants) versus murine TER119 RBCs in KitW41^−^ (*left dot plot*) and KITW41^+^ (*right dot plot*) chimeras after gating on alive hCD45^−^ mLy5.2^−^ cells. **b** Representative plot shows co-expression of CD71 relative to the CD235a marker in TER119^−^ Ly5.2^−^ (murine leukocyte markers) CD45^−^ (human leukocyte marker) cells. **c** Numbers of human CD235a^+^ RBCs among total RBCs and CD71^+^ CD235a^+^ cells among total human CD235a^+^ RBCs per 10^7^ in BM per femur of HIS-HEry chimeras. Each dot corresponds to one individual mouse (*n* = 17; Mean ± SEM are: 1.67 ± 1.23 for human CD235a + cells and 1.29 ± 0.97 for CD71^+^ CD235a^+^ cells). Source data are provided as a Source Data file. **d** Mean numbers of human CD235a^+^ RBCs per ml of HIS-HEry PB, untreated (Clod − ) or treated with clodronate liposome (Clod + ) (*n* = 4 mice in each group). Source data are provided as a Source Data file.

CH1-2hSa mice are alymphoid, RAG-deficient, and γc-deficient mice that express human signal regulatory protein receptor-alpha (SIRPα). SIRPα binds to the “don’t eat me” signal regulatory protein, CD47. In these mice, SIRPα is expressed under the *CSF1R* promoter. In addition, HLA-A2 and HLA-DR1 molecules were engineered to replace the murine MHC expression^9^. When neonatal CH1-2hSa mice were intra-hepatically engrafted with human cord blood (CB) CD34^+^ cells, they produced robust levels of human leukocytes with approximately 30% of PB leukocytes being of human origin within 4–5 months after engraftment^9^. In addition, we showed that CH1-2hSa mice could mount a functional human HLA-restricted T-cell immune response. However, virtually no human erythrocytes (CD235a^+^) were present in the BM of the chimeras (Fig. 1a, left panel, highlighted quadrant). This result was consistent with the impaired erythropoiesis previously described in other humanized mice models^10^. To improve human RBC production in CH1-2hSa chimeras, we introduced the hypomorphic KITW41 allele in the murine Stem Cell Factor Receptor (SCF-R or cKit), which is expressed on the surface of HSPCs. This allele contains one single nucleotide polymorphism that decreases SCF binding affinity, which is required for HSPC survival, growth, and differentiation^11^. By decreasing murine hematopoietic cell fitness and providing hematopoietic niches for human engrafted CD34^+^ cells^10,12,13^, cKITW41 expression dramatically increased the percentage of CD235a^+^ human red cells in the BM of CH1-2hSa chimeras (Fig. 1a, right panel, highlighted quadrant). This elevation in human CD235a^+^ RBCs was associated with a robust human erythropoiesis in the chimeric environment as on average, 11 million human RBCs cells were detected in the chimeric BM, which indicated that 25% of the total RBCs were of human origin (Fig. 1c and Supplementary Fig. 1a). As expected for developing erythrocytes, on average 85% of the human BM CD235a^+^ erythroid cells (around 9 million cells per femur) expressed CD71 (Fig. 1b, c and Supplementary Fig. 1a), a receptor that plays a key role in *P. vivax* RBC invasion^7^. In addition, the CD235a and CD71 co-expression profile was consistent with the different erythroid subsets observed in human BM (Fig. 1b, Supplementary Fig. 1a)^14^. The physiological maturation of human RBCs in the murine environment was further confirmed by the relative expression levels of CD49d and Band3 (Supplementary Fig. 1b), the induction of human adult hemoglobin expression (Supplementary Fig. 1c), and the observation that the human CD235a^+^ erythroid cells in the chimeric BM expressed various markers of erythropoiesis, namely: CD235a, CD36, CD44, CD234 (DARC; another entry receptor for *P. vivax*)^15^, and CD71 (Supplementary Fig. 1d). We called this new humanized mouse strain the Human Immune System Human Erythrocyte (HIS-HEry) model. In contrast to the BM compartment, the PB exhibited only low levels of human RBCs, most likely due to their destruction by peripheral murine macrophages, as described previously^16^ (Fig. 1d). The injection of clodronate liposomes, which depleted mouse macrophages, significantly raised the presence of human RBCs in the PB to approximatively 3% of the total circulating RBCs (Fig. 1d).

### HIS-HEry mice maintain intraerythrocytic *P. vivax* growth

**Fig. 2:**
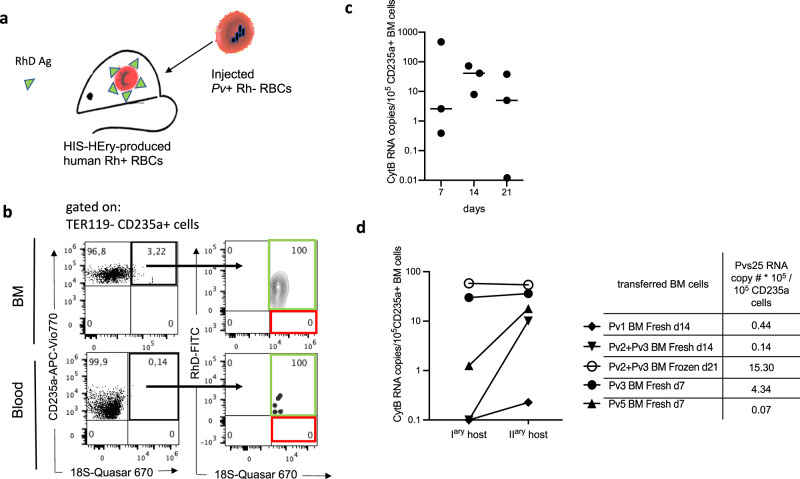
HIS-HEry chimeras support *P. vivax* infections. **a** HIS-HEry mice that produced human RhD^+^ RBCs (*green*) were infected with *P. vivax*-infected RhD^−^ RBCs from a patient (*red*). **b** Representative plots show the FlowFISH analyses of *P. vivax*-specific 18S rRNA expression in day-7-infected HIS-HEry mice. Cells were gated as alive TER119^−^ CD235a^+^. (*Left*) Upper and lower plots show the proportion of human CD235a^+^ 18S^+^ infected BM and PB cells (highlighted in black), respectively. (*Right*) The proportions of human endogenous RhD^+^ RBCs among the CD235a^+^ 18S^+^ RBCs are highlighted in green; the proportions of injected RhD^−^ RBCs among the CD235a^+^ RBCs are highlighted in red. **c** HIS-HEry chimeras *n* = 8 mice reconstituted with CD34^+^ cells from the same CB donor were infected with a mixture of *P. vivax* isolates (Pv2 + Pv3, see table in panel **d**). Plot shows the numbers of *P. vivax* CytB RNA copies per 10^5^ CD235a^+^ BM cells detected with qRT-PCR on days 7, 14, and 21 post-infection. Each dot represents one individual mouse. Mean CytB RNA copy numbers are: day 7 = 2.59, day 14 = 41.31, and day 21 = 5.01. Source data are provided as a Source Data file. This experiment is representative of 3 independent experiments. **d** Human BM RBCs from individual, infected HIS-HEry mice were adoptively transferred into secondary naive HIS-HEry recipient mice. Plot shows the numbers of *P. vivax* CytB RNA copy numbers per 10^5^ human BM RBCs cells, compared between donor (I^ary^ host) and secondary (II^ary^ host) hosts, at 7 days after transfer. The characteristics of the BM donor cells (i.e., the *Pv* isolate, the day post-infection, the fresh or frozen state, and the Pvs25 RNA copy numbers per 10^5^ human RBCs) are indicated in the table; *n* = 5 donor and 5 secondary host mice. Source data are provided as a Source Data file.

We next investigated whether CD71^+^ human reticulocytes from HIS-HEry mice could support *P. vivax* infection and multiplication. We used cryopreserved *P. vivax*-infected blood derived from patients in Brazil. Parasitemia levels ranged from 0.015% to 0.045% (Supplementary Table 1). To distinguish between host RBCs that developed within the HIS-HEry mice (‘endogenous’), and RBCs that were transferred from the original parasitized human donors, we took advantage of the differential allelic expression of Rhesus D (RhD) blood group markers. Thus, HIS-HEry mice that had been reconstituted with cord blood HPSCs from a RhD^+^ donor (Fig. 2a, green) were injected intravenously with thawed infected RBCs from a patient with RhD allele-negative (RhD^−^) blood (Fig. 2a, red). Mice were injected with clodronate liposomes throughout the experiment. *P*. *vivax* infections in the endogenous RhD^+^ RBCs and in the injected RhD^−^ RBCs were monitored with a new Flowcytometric RNA fluorescence in-situ hybridization (FlowFISH) method recently described^17^. This method allowed us to detect simultaneously the RhD surface expression on RBCs and their infection status, through *P. vivax* 18 S rRNA expression. On day 7 post-infection, 3.22% and 0.14% of human RBCs were parasitized in the BM and PB of chimeric mice respectively (Fig. 2b). Remarkably, at this time, virtually all infected RBCs, including nucleated RBCs, were derived from the humanized host mice (RhD^+^) and not from the parasitized human donor (RhD^−^; Fig. 2b and Supplementary Fig. 2a). By staining BM cells from infected mice with Giemsa stain, we confirmed the presence of parasites in nucleated RBCs (Supplementary Fig. 2b).

We then assessed whether *P. vivax* infections in HIS-HEry mice could be maintained for extended periods of time. We measured *P. vivax*-specific transcript levels over time in the BM of individual infected mice that had been reconstituted with the same CB cells and infected with the same mixture of *P. vivax* isolates (Pv2 + Pv3). *P. vivax* RNA levels were variable within the experimental group of mice, but 8 of 9 mice remained infected with stable average RNA levels for 21 days post-infection (Fig. 2c).

Importantly, the adoptive transfer of fresh or frozen BM cells from previously infected mice into naive secondary HIS-HEry mice could establish a new infection (Fig. 2d). We used fresh and frozen BM samples collected at various time points after injecting HIS-HEry mice with different isolates. Four out of five secondary transfers resulted in increased *P. vivax* parasitemia levels at 7 days after the inoculation (based on the transcripts levels of the housekeeping gene, cytochrome B (CytB)), compared to levels in the original inoculated BM cells. The most striking increase after transfer was observed upon the injection of donor BM, which presented undetectable parasitemia, based on CytB RNA levels (Fig. 2d). In addition, we found that parasite expansion was inversely correlated with the levels of Pvs25 transcripts (a specific marker of gametocytes) detected in donor cells prior to injection into the secondary host mice (Fig. 2d). These results demonstrated that in vivo propagation of *P. vivax* blood stages was feasible in a murine model.

### De novo *P. vivax* gametocyte differentiation in HIS-HEry mice

**Fig. 3:**
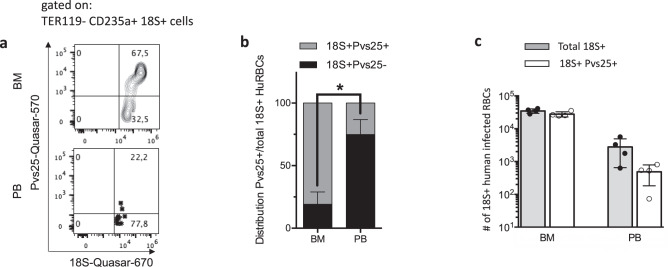
HIS-HEry chimeras support de novo *P. vivax* sexual differentiation. HIS-HEry mice were infected with the Pv1 isolate, and RBCs were examined at 7 days post-infection. **a** Representative FlowFISH plots show the proportions of *P. vivax* gametocyte-specific, Pvs25 RNA expression in all gated TER119^−^ CD235a^+^ *P. vivax* 18S^+^ (highlighted parasitized human cells in the BM (*upper panel*) and in the PB (*lower panel*). **b** Distribution of Pvs25^+^ 18S^+^ gametocytes (*gray bars*) versus Pvs25^−^ 18S^+^ asexual blood stages (*black bars*), among BM and PB human CD235a^+^ RBCs; *n* = 4 individual mice. Two-tailed paired Student’s *t*-test was performed to compare the representation of Pvs25- 18S + RBCs between the BM and PB samples. *P-value= 0.0004; Mean ± SDs are: BM Pvs25- mean = 19.13 ± 17.5; PB Pvs25- mean = 74.83 ± 11.94. **c** Mean numbers of total 18 S + infected RBCs (*open bars*) and Pvs25 + 18 S + gametocytes (*gray bars*) in the BM (per femur) and in PB (per ml). *n* = 4 individual mice (same as in **b**). Mean ± SDs are: BM total 18 S + = 34,793 ± 5367; BM Pvs25 + 18 S + = 27,815 ± 4968; PB total 18 S + = 2786 ± 2132; PB Pvs25 + 18 S + = 484 ± 302. Source data are provided as a Source Data file.

To test whether sexual differentiation of *P. vivax* occurred in HIS-HEry chimeras, we performed FlowFISH analysis to quantify the distribution of gametocytes versus asexual parasites in both the BM and PB from HIS-HEry mice. In this analysis, we used RNA probes that specifically recognized Pvs25^+^ mature gametocytes^18^ (Fig. 3a). We found that, at 7 days post-infection, the proportion of 18S^+^ Pvs25^+^ gametocytes was larger in the BM than in the PB (e.g., Fig. 3a). This difference was highly significant (*P* = 0.0004); the average distributions were 64% ±17 gametocytes in the BM and 8% ±9 gametocytes in the PB (Fig. 3b).

Next, we determined the numbers of parasitized cells. We found an average of 34,793 ± 5367 total parasitized cells (18S rRNA^+^) per femur, and among those, 27,815 ± 4968 were Pvs25 RNA^+^ gametocytes. In the PB, we found about 2786 ± 2132 total parasitized cells (18 S rRNA^+^) per ml, and among those, 484 ± 302 were Pvs25 RNA^+^ gametocytes (Fig. 3c). Given that virtually none of the infected RBCs detected on day 7 post-infection originated from the injected isolate on day 7 post-infection (Fig. 2b), we concluded that sexual commitment had occurred in the human erythrocytes produced de novo in the chimeric mice. This conclusion was supported by the FlowFISH experiment, where Pvs25^+^ 18S^+^ gametocytes were detected within RhD^+^ CD235a^+^ BM RBCs on day 7 post-infection (Supplementary Fig 2a, lower row). Of note, the observation that BM nucleated RBCs (DAPI^+^) were infected with gametocytes (Supplementary Fig. 2a) indicated that erythroblasts could support both asexual parasite invasion and sexual differentiation. Furthermore, these data supported the notion that gametocytes preferentially resided within the BM in our mouse model, similar to previous findings reported in human studies^19^.

### *P. vivax* gametocyte transmission and sporozoite formation

**Fig. 4:**
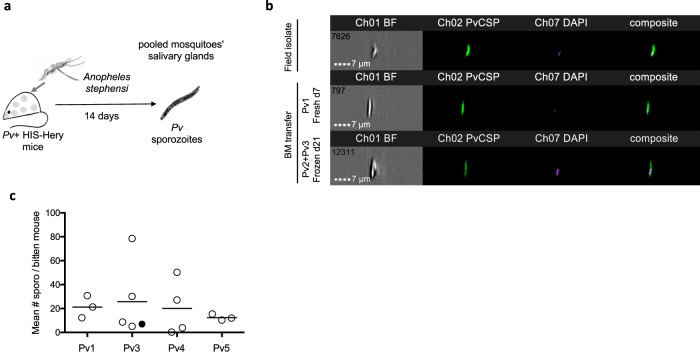
HIS-HEry chimeras support *P. vivax* sexual differentiation, gametocyte transmission to *Anopheles*, and sporozoite formation. **a** Individual HIS-HEry infected chimeras were bitten by *Anopheles stephensi* mosquitoes, and sporozoite production in pooled mosquito salivary glands was assessed 14 days later. **b** Representative FlowFISH ImageStream profiles of salivary gland sporozoites, stained with a fluorescent marker for *P. vivax* circumsporozoite protein (PvCSP, *green*; Chanel 2 for FITC) and DAPI to visualize nuclei (Chanel 7). The composite images (right column) show colocalization of nuclei and surface PvCSP. The different samples are from: (*upper row*) frozen patient field blood isolates, (*middle row*) mosquitoes that fed on day-7 Pv1-infected HIS-HEry mice, and (*bottom row*) mosquitoes that fed on day-7 HIS-HEry mice infected with frozen BM isolated from day-21-infected mice (see Fig. 2b). **c** Plots show the mean numbers of sporozoites obtained per mosquito fed on individual mice(sporo; calculated as the total sporozoites isolated from all mosquitoes that bit each individual mouse/the total number of mosquitoes that bit that individual mouse—see Supplementary Fig. 3b), for each group of mice infected with the indicated Pv isolates. The filled dot in the Pv3 column indicates that this mouse was infected with a pool of Pv2 + Pv3. Each dot represents one individual mouse. *n* = 15 individual mice dispatched according the isolates used to infect. Means ± SD are: Pv1 = 21.2 ± 9.24; Pv3 = 31.1 ± 23.2; Pv4 = 20.12 ± 23.21; Pv4 = 12.37 ± 2.59. Source data are provided as a Source Data file.

Given the presence of gametocyte stages in the PB of the *P. vivax*-infected HIS-HEry mice, we aimed to assess their viability and maturation by performing transmission assays. HIS-HEry mice infected with different *P. vivax* isolates were used to feed *Anopheles stephensi* mosquitos. The dissection of mosquito midguts allowed us to visualize oocysts in mosquitoes that had fed on the infected HIS-HEry mice (Supplementary Fig. 3a). Fourteen days after exposure to mice, salivary glands from pooled mosquitoes (i.e., 30–60 mosquitoes per mouse) were dissected (Fig. 4a), and the presence of sporozoites was analyzed and counted in a FlowFISH assay with specific antibodies against the major surface circumsporozoite protein (CSP) (Fig. 4b, c and Supplementary Fig. 3b). Although only few dissected mosquitoes showed detectable oocysts numbers in the midgut (Supplementary Fig. 3a), sporozoites were found in mosquito salivary glands from 14 out of 15 infected HIS-HEry chimeras (Fig. 4c). This result showed the high efficiency of the chimeras at supporting gametocyte transmission to mosquitoes via blood feeding. The average numbers of sporozoites per mosquito that fed on an individual mouse (Fig. 4c) were deduced from the total number of sporozoites obtained from the pooled mosquitoes that had bitten individual mouse (see Supplementary Fig. 3b). These average numbers varied from 12.37 to 25.68 sporozoites per mosquito per mouse, depending on the *P. vivax* isolates used to infect each group of mice. Moreover, these numbers varied widely among the individual mice from each group, with an SD of 2.56 to 31.09. The total numbers of sporozoites derived from individual mice also varied widely, ranging from 147 to 3000 sporozoites per mouse (Supplementary Fig. 3b). Importantly, gametocytes could be transmitted to mosquitoes even from HIS-HEry mice with low CytB RNA levels (3–75 copies/ml of PB; Supplementary Fig. 3b, see mice infected with Pv1 and Pv4). In addition, sporozoites were obtained from mosquitoes that had bitten secondary host mice that had been infected with frozen BM cells from 21 day-infected donor mice (Supplementary Fig. 3b, mouse #4). That finding validated the model for long-term transmission experiments with the same clinical isolates.

## Discussion

The generation of a novel humanized mouse model that can produce high levels of human erythropoiesis overcomes a major obstacle in malaria research. We explored the potential of HIS-HEry humanized mice for maintaining in vivo infections of *P. vivax*. We achieved *P. vivax* blood-stage growth and proliferation in chimeric mice for at least 3 weeks after infection. However, we assume that infections could be maintained for longer times, because we found that HIS-HEry chimeras survived for over one year keeping high levels of hematopoiesis and erythropoiesis during that time. Both these features relied on the *KITW41* mutant allele expression, which obviates detrimental irradiation prior to HSPC transplantation^20^ and increases human HSPC maintenance in the chimeric BM^8^.

Our HIS-HEry mouse model infected with clinical *P. vivax* isolates mimicked several of the important features observed in *P. vivax*-infected humans. First, both sexual and asexual *P. vivax* blood stages were detected in the BM, as described in BM from patient biopsies^19^. Second, the high levels of sexually committed parasites found in BM, compared to the PB, were consistent with previous observations in BM biopsies from *P. vivax*-infected patients^19^. That finding supports the hypothesis that the BM may represent a niche and reservoir for sexual-stage parasites. Interestingly, our preliminary experiments showed that salivary gland sporozoites isolated from mosquitoes that had fed on BM cells from parasitized HIS-HEry mice could be used to infect a hepatocytic cell line in vitro (C. Luiza-Batista and S. Thiberge, unpublished data). Our results show for the first time that BM harbor mature transmissible gametocytes, an observation consistent with the high transmission rate observed in *P. vivax* malaria^21^. Third, we found that de novo gametocytes were generated within a few days after mouse infection. This result confirmed that sexual differentiation occurred faster in *P. vivax* infections than in *P. falciparum* infections^3^. Fourth, using a new FlowFISH method that we recently devised^17^, we unequivocally demonstrated that nucleated human RBCs could be infected in vivo (Supplementary Fig. 2a). That finding confirmed previous observations based on transmission electron microscopy of BM biopsies from patients^22^. Fifth, HIS-HEry mice with low PB parasite densities could transmit parasites to *Anopheles* mosquitoes. This finding recapitulated the observation that patients with very low parasitemia could contribute to human-to-mosquito transmission, and consequently, they may constitute an important infectious reservoir^23^. Therefore, and in contrast with current mouse model for human *Plasmodium* blood-stage infections, based on the adoptive transfer of human mature RBCs (for P*. falciparum*^24^) or reticulocytes (for *P. vivax*^25^), the HIS-HEry model provides unique opportunities for addressing crucial questions associated with BM infections, such as parasite-induced dyserythropoiesis, which leads to anemia, and more generally, problems linked to BM hematopoietic disorders that may be associated with parasite-mediated inflammation^5^.

Taking advantage of the finding that parasites were localized in the BM, we established a robust protocol for transferring fresh or frozen infected BM cells derived from HIS-HEry mice into a secondary uninfected mouse to produce a *P*. *vivax* infection. We observed a strong increase in parasite load upon the secondary transfer in 3/5 secondary hosts. This increase was related to low sexual parasite representation among the transferred parasites and to the probable expansion of asexual parasites. Alternatively, this increase may rely on the selective survival of clinical isolates that were better adapted to the murine host environment, as previously shown for *P*. *falciparum*^26^. We hypothesize that repeated passages in HIS-HEry mice possibly will establish *P. vivax* lines with higher parasitemia that could provide sufficient biomass for biochemical studies or even to establish transfected of *P*. *vivax* lines. In addition to providing a method for amplifying and storing *P. vivax* lines, the HIS-HEry mice will facilitate explorations of novel anti-malarial interventions and the elimination of this pathogen in the BM niche. Of note, the average amount of parasitized cells obtained from one femur (see Fig. 2a) could be readily increased by harvesting more bones, since femur represents only 10% of the total BM cell content^27^. Thus, sufficient material could be harvested for in depth molecular characterizations of the parasite development in the BM environment using singlecell sequencing analysis of the infected host cells.

The vast majority of infected HIS-HEry mice was able to transmit *P. vivax* to *Anopheles*, which led to the production of salivary gland sporozoites. Transmission was possible with cells from mice with low parasitemia and with cells collected at 21 days after the infection. Currently, the experimental generation of *P. vivax* sporozoites requires fresh blood from patients infected with *P. vivax*, for feeding to mosquitoes in *P. vivax* endemic regions^28^. It was previously shown that 2-100 sporozoites from mosquitoes fed with blood from patients infected with *P. vivax* were sufficient for productive infections in human volunteers^29^. Accordingly, our HIS-HEry mice could provide sufficient numbers of sporozoites for conducting experimental infections in humans. The combination of primary human liver cell cultures and *P. vivax* sporozoites will provide open new avenues of research on this neglected, but important topic. In particular, studies are needed on hypnozoite forms, which remain a major hurdle to eradicating this disease^2^.

Although this work undoubtedly represents a major breakthrough for the study of *P. vivax*, the model may be further improved, notably, regarding the low number of circulating human RBCs and the low number of sporozoites produced upon gametocyte transmission when mosquitos feed on HIS-HEry mice. Indeed, although the BM of HIS-HEry mice supports long-term human erythropoiesis, peripheral accumulation of human RBCs is impeded, due to their elimination by murine macrophages^16^. The use of liposome clodronate treatment to eliminate host mouse macrophages allowed us to increase the levels of peripheral human RBCs but human blood RBCs remain low, which probably limits the numbers of gametocytes transmissible to mosquitoes. Consequently, most of the parasite biomass is restricted to the BM of the chimeric mice. Of note, we did not observe any major impact of clodronate on human hematopoiesis, neither on parasites. Based on a recent study, gadolinium chloride injections might be a good alternative to clodronate injections for macrophage depletion, because gadolinium chloride is less toxic to animals^30^. Moreover, the use of Cobra Venom Factor, or a genetic approach for targeting mouse complement C3, was shown to increase survival of human RBCs in mouse blood^30,31^. This approach could present an advantage, because it could obviate the effects of non-specific macrophage depletion on human immunity in infected chimeric mice. Finally, in addition to increasing circulating human RBCs in HIS-HEry mice, transmission efficiency and sporozoite production might be increased by using other *Anopheles* species that are known to naturally transmit *P. vivax* in endemic regions. For example, *A. darlingii* is the prevalent transmission vector for *P. vivax* in Brazil.

In conclusion, we describe a new model of humanized mice that was productively infected with *P. vivax*. Moreover, this model reproduced several key features of natural infections, including the settlement of sexual parasites in a BM niche and parasite transmission in low parasitemia settings. This model holds promise for understanding in vivo host-parasite interactions. Moreover, despite the fact that it requires optimization, the HIS-HEry mouse model could offer a unique opportunity for in vivo testing of novel anti-malarial interventions. As this mouse model expresses human HLA and has a functional associated T-cell immune response^9^, it will facilitate testing new vaccine strategies against asexual and sexual parasite stages. Additionally, it will provide a platform for drug screening in vivo. Importantly, this model also holds promise for devising new transmission-blocking strategies, in both vertebrate and invertebrate hosts.

## Methods

### Generation of Rag2^tm1Fwa^ IL2rgt^m1Cgn^ B2m^tm1Unc^ H2-Ab1^tm1Doi^ Tg(HLA-DRA*0101,HLA-DRB1*0101)1Dma Tg(HLA-A2) Tg(SIRPA) Hc^0^ cKitW41^d/d^ (CH1-2hSaW41) host mice

Procedures involving mice were previously approved by local Animal Ethics Committees (CETEA #089) and registered with the French authorities #15341-2018053115015969. CH1-2hSa mice generated in our laboratory^9^ were crossed with B6W41 mice (generously provided by Claudia Waskow, University of Dresden, Germany) to generate CH1-2hSaW41 mice. All strains were on the C57Bl/6 background. Mice were bred and maintained in dedicated facilities of the Institut Pasteur with provided food and water ad libitum.

### Isolation of CD34 + human cord blood HSPCs and transplantation into CH1-2hSaW41 host mice

Human cord blood samples were obtained from de-identified healthy donors (AP-HP, Hôpital Saint-Louis, Unité de Thérapie Cellulaire, CRB-Banque de Sang de Cordon, Paris, France—authorization number: AC-2016-2759). Mononuclear cells were enriched by centrifugation with Ficoll Hypaque. Next, CD34^+^ cells were selected with immunomagnetic beads in an AutoMACS pro instrument (Myltenyi Biotec), and the purity (>95%) was checked Flow cytometry. RhD expression levels were determined with flow cytometry (see below). Alternatively, human cord blood CD34 + cells were purchased from AbcellBio company, France.

To generate HIS-HEry chimeras, 1–4-day-old CH1-2hSaW41 neonates were subjected to liver injections of 10-30,000 human cord blood CD34^+^ cells in 30 µl phosphate-buffered saline (PBS) with a 30-gauge needle (Becton Dickinson), as previously described. For some experiments, mice were anesthetized, then injected with 200 µl clodronate liposomes. One day later, blood samples were collected to assess the RhD status of the human RBCs with Flow cytometry (see Flow cytometry section).

### Flow cytometry

To analyze the surfaces of human erythrocytes, cells were stained with monoclonal antibodies (mAbs) against Ly5.2, CD45, Ter119, CD235a, CD49d, Band3, CD71, CD234 (DARC), or CD44 or with anti-RhD antibodies. Nuclei were stained with DAPI. Secondary anti-human IgG1 mAbs were used to reveal RhD staining.

For adult HbB and fetal HbF hemoglobin expression, cells were first stained with antibodies against Ly5.2, CD45, TER119, and CD235a. Next, cells were fixed for 30 min in 500 µl of 4% paraformaldehyde (Thermo Scientific™) in PBS and 0.0075% glutaraldehyde (Electron Microscopy Sciences). For dead cell exclusion, cells were stained with the LIVE/DEAD™ Fixable Dead Cell Stain Sampler Kit(Invitrogen), according to the manufacturer’s instructions, or with 7-aminoactinomycin D (Sigma). After permeabilization with 1 mM Triton, cells were co-stained with anti-HbB and anti-HbF mAbs and washed twice with PBS. Cells were processed on a BD LSRFortessa™ with the FACSDiva Software version 8.0.1 (BD Biosciences Becton Dickinson) (BD Biosciences, Becton Dickinson), and the data were analyzed with FlowJo LLC software v10.6.1 (Becton Dickinson). All antibodies used are listed in Supplementary Table 2. The gating strategy is detailed in Supplementary Figure 4.1. Supplementary Figure 4a.

### *P. vivax* isolates

Study protocols were approved by the Institutional Review Board of the Institute of Biomedical Sciences, University of São Paulo, and by the National Human Research Ethics Committee of the Ministry of Health of Brazil (CAAE: 64767416.6.0000.5467); all patients provided written informed consent. RhD expression was determined for each isolate (see the flow cytometry section). Seven clinical *P. vivax*-infected blood samples were collected in northwestern Brazil in the context of an ongoing cohort study (ClinicalTrials.gov, NCT03689036). These *P. vivax* isolates (Pv) were leukocyte-depleted and cryopreserved in liquid nitrogen, as described previously^32^ (Supplementary Table 1).

### HIS-HEry mouse infection with *P. vivax*

*P. vivax* isolates were thawed with a stepwise NaCl method, as previously described^33^, and washed in PBS. Before being used to infect mice, the isolates were tested for *Plasmodium* parasitemia by examining thick PB smears with Giemsa staining. Next, RBC pellets of the isolates were resuspended in PBS at a concentration of 2.5.10^6^ to 5.10^6^ parasitized RBCs per ml. One day before infection, 12- to 20-week-old HIS-HEry chimeras received intravenous injections of 200 μl liposome clodronate (Liposoma BV, The Netherlands). This injection was repeated every 5 days for the duration of the experiments. HIS-HEry mice were infected intravenously, under anesthesia, with 10^6^−5.10^6^ infected RBCs from either a single isolate (Pv1, Pv2, Pv3, or Pv4) or a mixture of three isolates (Pv5), except when stated otherwise in figure legends. Then, BM and blood were collected from euthanized mice on days 1, 7, 14, or 21 after infection. To collect BM, the two femurs, tibias, and humeri were dissected, the bones were crushed, and cell suspensions were filtered through 40-μM filters before processing. Each experiment was conducted with chimeras engrafted with CD34^+^ cells from the same CB donor.

### *P. vivax* RNA quantification and characterization with qRT-PCR

After freezing on dry ice, PB and 10^6^ BM cells were pelleted and resuspended in TRIzol LS reagent, followed by RNA extraction, performed as described by the manufacturer (Invitrogen, Carlsbad, CA). Then, cDNA was synthesized in a 20 μl solution with the single-tube procedure provided in the SuperScript™ II Reverse Transcriptase cDNA synthesis kit (Invitrogen Carlsbad, CA), according to manufacturer instructions. Quantitative PCR (qPCR) was performed on 2 μl cDNA with the SsoAdvanced™ Universal SYBR^®^ Green Supermix (Bio-Rad) and the following primer pairs: CytB-Forward: 5′-TGGAGTGGATGGTGTTTTAGA 3′ and CytB-Reverse: 5′ TTGCACCCCAATAACTCATTT-3′; Pvs25-Forward: 5′-AACGAAGGGCTGGTGCACCTTT-3′ and Pvs25-Reverse: 5′-AGCAACCTGCACTTTGGATTTCCG-3′. In parallel, different dilutions of a plasmid solution containing 1 copy of *Plasmodium* CytB and 10^2^ to 10^5^ copies of Pvs25 were amplified in the same conditions to set up a standard curve. The data were analyzed with Bio-Rad CFX Maestro Version 4.1.2434.232. Results are expressed as the number of CytB or Pvs25 RNA copies per 10^5^ human BM CD235a^+^ RBCs, derived from a FACS analysis of the % of CD235a^+^ cells.

### *P. vivax* quantification and sexual characterization with FlowFISH analysis

We recently described the FlowFISH method, which was adapted from the commercial method provided by Stellaris®^17^. Briefly, both PB and BM were first stained with the LIVE/DEAD™ Fixable Blue Dead Cell Stain Kit (Invitrogen). Next, cells were stained with conjugated mAbs against TER119, CD235a, CD71, and in some experiments, non-conjugated anti-RhD mAbs, followed by FITC-conjugated anti-human IgG1. Stained cells were fixed for 30 min in 500 µl of a 4% paraformaldehyde solution (Thermo Scientific™) in PBS and 0.0075% glutaraldehyde (Electron Microscopy Sciences) at room temperature (RT). After washing twice with PBS, fixed cells were hybridized, according to manufacturer instructions (www.biosearchtech.com/stellarisprotocols), with a set of RNA Stellaris FISH Probes (Biosearch Technologies); one set of probes was specific for *P. vivax* 18 S rRNA (the sequences of the probes are unknown as they were designed by Biosearch Technologies) and was conjugated to quasar-670; the other set of probes was specific for *P. vivax* Pvs25 RNA (the sequences are described in Supplemental Table 3) and was conjugated to quasar-570. The Pvs25 RNA probes were designed with the Stellaris® FISH Probe Designer (Biosearch Technologies), available online at: www.biosearchtech.com/stellarisdesigner. Importantly, the *P*. *vivax* 18S probes did not cross-react with mouse or human 18S rRNA. For the flow imaging procedure, the hybridized RBCs were stained with DAPI, as described previously. Cells were processed on a BD LSRFortessa™ with the FACSDiva Software version 8.0.1 (BD Biosciences Becton Dickinson), and data were analyzed with FlowJo LLC software, v10.6.1 (Becton Dickinson). The gating strategy is detailed in Supplementary Fig. 4.2.

For sporozoite detection, salivary glands were collected from fed *Anopheles* mosquitoes, dilacerated, and filtered through a 35-µm mesh. After washing in PBS, recovered sporozoite cells were stained with anti-PvCSP mAbs (1:500 dilution) and fixed for 10 min in 100 µl of a 2% paraformaldehyde solution (Thermo Scientific™) in PBS. Sporozoites were then stained with DAPI, as described above. Sporozoites were analyzed with an Image stream ISX MkII flow cytometer (Amnis Corp, EMD Millipore), with channel 01 for BrightField, channel 02 for FITC-conjugated anti-PvCSP mAb detection, and channel 07 for DAPI detection. Data were analyzed with Luminex corp Software IDEAS version 6.2. All antibodies used are listed in Supplementary Table 2.

### Secondary transfers of BM cells from infected HIS-HEry mice into naive HIS-HEry chimeras

BM human CD235a + cells collected from infected HIS-HEry chimeras were first enriched with an AutoMACS pro. Then, they were sorted with  a MoFlo Astrios Cell Sorter (Beckman Coulter). Next, cells were either directly injected intravenously into clodronate liposome-treated, secondary naive HIS-HEry mice, or they were frozen in 50% IMDM 50% freezing solution (SVF 20% DMSO). At later times, the frozen cells were thawed and intravenously injected (10,000 to 120,000 cells) into clodronate liposome-treated, secondary naive, HIS-HEry mice.

### Mosquito feeding

Female *Anopheles stephensi* were either purchased from Radboud University Medical Center (RUMC), Nijmegen, The Netherlands, or they were reared in the Center for Production and Infection of A*nopheles* (CEPIA) at the Institut Pasteur, with standard procedures (SDA500 strain). HIS-HEry infected mice were anesthetized intraperitoneally with approximately 70 mg/kg ketamine and 2 mg/kg xylazine before they were transferred to the BSL-3 facility in CEPIA. Anesthetized mice were placed on a mesh screen that covered a container of 30–60 4-7-day-old female mosquitoes. Mosquitoes were then allowed to feed for 10–15 min. Mice were then euthanized, and BM and PB were collected for further analysis. Blood feeding was confirmed by visualizing blood engorged female mosquitoes. Unfed mosquitoes were removed, and the fed mosquitoes were maintained at the CEPIA facility under standard breeding conditions. At day 14 post-feeding, salivary glands from pooled mosquitos were dissected, and sporozoites were identified on an Image stream ISX MkII flow cytometer (Amnis Corp, EMD Millipore) and quantified with FlowFISH analysis (version 10.6.1).

### Statistics

The mean ± SD values are indicated in figures and/or legends. The two-tailed paired Student’s *t*-test was performed to compare two groups (Fig. 2b). The *P*-values deemed significant are indicated in the figures. Statistical analyses were performed with Prism 9.0a software (GraphPad Software).

### Reporting summary

Further information on research design is available in the Nature Research Reporting Summary linked to this article.

## Supplementary information

Supplementary Information

Reporting Summary

## Data Availability

Data supporting the findings of this work are available within the paper and its Supplementary Information files. A reporting summary for this Article is available as a Supplementary Information file. The datasets analyzed during the current study are available from the corresponding author upon reasonable request. Source data are provided with this paper.

## Source data

Source Data

## References

[1] World Health Organization. World malaria report 2021—WHO. https://www.who.int/publications/i/item/9789240040496 (World Health Organization, 2021).

[2] Price RN, Commons RJ, Battle KE, Thriemer K, Mendis K (2020). *Plasmodium vivax* in the Era of the shrinking *P. falciparum* Map. Trends Parasitol..

[3] Merrick CJ (2021). Hypnozoites in Plasmodium: do parasites parallel plants?. Trends Parasitol..

[4] Obaldia, N. 3rd et al. Bone marrow is a major parasite reservoir in *Plasmodium vivax* infection. *MBio* **9**, 10.1128/mBio.00625-18 (2018). 10.1128/mBio.00625-18 PMC5941073 29739900

[5] Silva-Filho JL (2020). *Plasmodium vivax* in hematopoietic niches: hidden and dangerous: (trends in parasitology 36, 447-458, 2020). Trends Parasitol..

[6] Gunalan K, Rowley EH, Miller LH (2020). A way forward for culturing *Plasmodium vivax*. Trends Parasitol..

[7] Gruszczyk J (2018). Transferrin receptor 1 is a reticulocyte-specific receptor for *Plasmodium vivax*. Science.

[8] Sippel TR, Radtke S, Olsen TM, Kiem HP, Rongvaux A (2019). Human hematopoietic stem cell maintenance and myeloid cell development in next-generation humanized mouse models. Blood Adv..

[9] Serra-Hassoun M (2014). Human hematopoietic reconstitution and HLA-restricted responses in nonpermissive alymphoid mice. J. Immunol..

[10] Rahmig S (2016). Improved human erythropoiesis and platelet formation in humanized NSGW41 Mice. Stem Cell Rep..

[11] Broudy VC (1997). Stem cell factor and hematopoiesis. Blood.

[12] Cosgun KN (2014). Kit regulates HSC engraftment across the human-mouse species barrier. Cell Stem Cell.

[13] McIntosh BE (2015). Nonirradiated NOD,B6.SCID Il2rgamma-/- Kit(W41/W41) (NBSGW) mice support multilineage engraftment of human hematopoietic cells. Stem Cell Rep..

[14] Wangen JR, Eidenschink Brodersen L, Stolk TT, Wells DA, Loken MR (2014). Assessment of normal erythropoiesis by flow cytometry: important considerations for specimen preparation. Int. J. Lab. Hematol..

[15] Horuk R (1993). A receptor for the malarial parasite *Plasmodium vivax*: the erythrocyte chemokine receptor. Science.

[16] Hu Z, Van Rooijen N, Yang YG (2011). Macrophages prevent human red blood cell reconstitution in immunodeficient mice. Blood.

[17] Luiza-Batista, C. et al. Flowcytometric and ImageStream RNA-FISH gene expression, quantification and phenotypic characterization of blood and liver stages from human malaria species. *J. Infect. Dis.*10.1093/infdis/jiab431 (2021). 10.1093/infdis/jiab431 PMC9071310 34453537

[18] Sa JM, Cannon MV, Caleon RL, Wellems TE, Serre D (2020). Single-cell transcription analysis of *Plasmodium vivax* blood-stage parasites identifies stage- and species-specific profiles of expression. PLoS Biol..

[19] Baro B (2017). *Plasmodium vivax* gametocytes in the bone marrow of an acute malaria patient and changes in the erythroid miRNA profile. PLoS Negl. Trop. Dis..

[20] Waskow C (2009). Hematopoietic stem cell transplantation without irradiation. Nat. Methods.

[21] Douglas NM (2013). Gametocyte dynamics and the role of drugs in reducing the transmission potential of *Plasmodium vivax*. J. Infect. Dis..

[22] Ru YX (2009). Invasion of erythroblasts by Pasmodium vivax: A new mechanism contributing to malarial anemia. Ultrastruct. Pathol..

[23] Alves FP (2005). Asymptomatic carriers of Plasmodium spp. as infection source for malaria vector mosquitoes in the Brazilian Amazon. J. Med. Entomol..

[24] Tyagi RK (2018). Humanized mice are instrumental to the study of *Plasmodium falciparum* infection. Front. Immunol..

[25] Schafer C (2020). A humanized mouse model for *Plasmodium vivax* to test interventions that block liver stage to blood stage transition and blood stage infection. iScience.

[26] Jimenez-Diaz MB (2009). Improved murine model of malaria using *Plasmodium falciparum* competent strains and non-myelodepleted NOD-scid IL2Rgammanull mice engrafted with human erythrocytes. Antimicrob. Agents Chemother..

[27] Colvin GA (2004). Murine marrow cellularity and the concept of stem cell competition: geographic and quantitative determinants in stem cell biology. Leukemia.

[28] Collins KA (2020). A *Plasmodium vivax* experimental human infection model for evaluating efficacy of interventions. J. Clin. Invest.

[29] Herrera S (2011). Consistent safety and infectivity in sporozoite challenge model of *Plasmodium vivax* in malaria-naive human volunteers. Am. J. Trop. Med. Hyg..

[30] Yamaguchi T (2021). Generation of novel human red blood cell-bearing humanized mouse models based on C3-deficient NOG mice. Front. Immunol..

[31] Chen B (2017). Complement depletion improves human red blood cell reconstitution in immunodeficient mice. Stem Cell Rep..

[32] de Oliveira TC (2017). Genome-wide diversity and differentiation in New World populations of the human malaria parasite *Plasmodium vivax*. PLoS Negl. Trop. Dis..

[33] Saunders GM, Talmage DW, Scott V (1948). The use of *Plasmodium vivax* preserved by freezing in inducing malaria. J. Lab. Clin. Med..

