# Supplementary material for: Humanized mice for investigating sustained Plasmodium vivax blood-stage infections and transmission
Source: Nat Commun. 2022 Jul 15;13:4123. doi: 10.1038/s41467-022-31864-6 (PMC9287384; doi:10.1038/s41467-022-31864-6)
Supplement: Supplementary file 1 — Supplementary Information [file 41467_2022_31864_MOESM1_ESM.pdf]

Humanized mice for investigating sustained *Plasmodium vivax* blood-stage infections and transmission

Supplementary information

# *Pv* isolate	parasitemia (%)	# parasites injected per mouse
Pv1	0.045	9×10^5
Pv2	0.031	6.2×10^5
Pv3	0.023	4.6×10^5
Pv4	0.015	3×10^5
Pv5.1<br>Pv5.2 } Pv5<br>Pv5.3	0.044<br>0.030 } 0.035<br>0.031	2.9×10^5<br>1×10^5 } 4.9×10^5<br>1×10^5

Supplementary Table 1| *P. vivax* (Pv) isolates from Acre (Brazil) used to experimentally infect HIS-HEry chimeric mice. Mice were inoculated with either a single isolate (Pv1, Pv2, Pv3, or Pv4) or a mixture of 3 isolates (Pv5), except when stated otherwise in figure legends. The percent parasitemia was measured with Giemsa-stained blood smears and microscopy. The number of infected blood cells in each inoculum are shown for each isolate, except for Pv5; for Pv5, the brackets indicate averages of the individual isolates. All isolates tested negative for *P. falciparum* with qPCR.

Supplementary Fig. 1

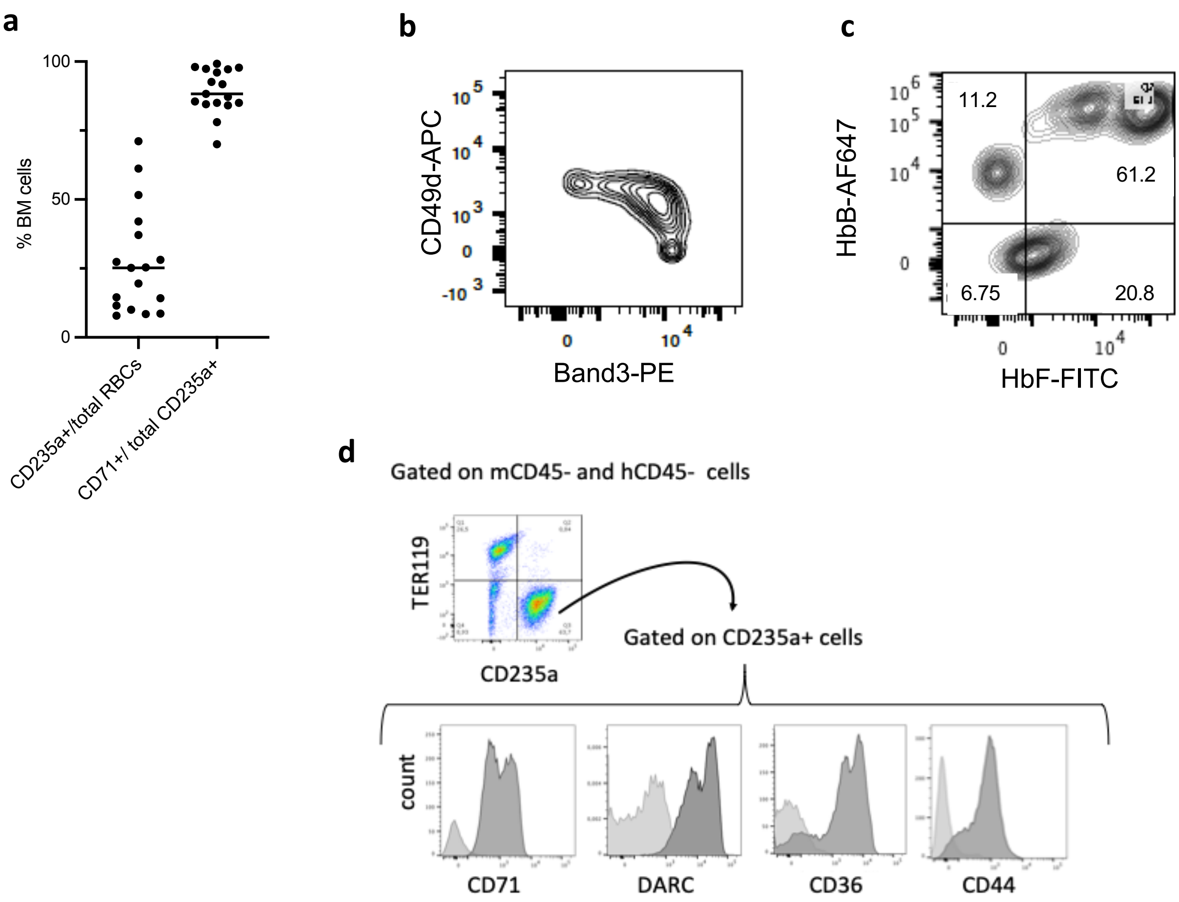

Supplementary Fig. 1 | HIS-HEry chimeras support efficient human erythropoiesis. BM human erythrocytes (RBCs) were isolated from 16- to 24-week-old HIS-HEry chimeras. Then, the RBCs were analyzed by Flow cytometry. **a**, Distributions of (*left*) human CD235^{+} RBCs among total mouse RBCs, and (*right*) the percentage of total CD71^{+} cells among total human CD235^{+} RBCs from HIS-HEry chimeras (n=17). Source data are provided as a Source Data file. **b**, Flow cytometry results show CD49d and Band3 co-expression levels in CD235^{+} human BM RBCs. **c**, Flow cytometry results show intracellular co-expression of mature human hemoglobin subunit beta (HbB) and fetal hemoglobin (HbF) in human RBCs in HIS-HEry chimeras.

d, (*Upper panel*) Flow cytometry results show the isolation of human TER119^{-} CD235^{+} BM RBCs. (*Lower panel*) Representative histograms show the expression of CD71, DARC (CD234), CD36, and CD44 (dark gray) in RBCs from HIS-HEry chimeras, compared to their expression in RBCs from control non-reconstituted mice (light gray).

Supplementary Fig. 2

a

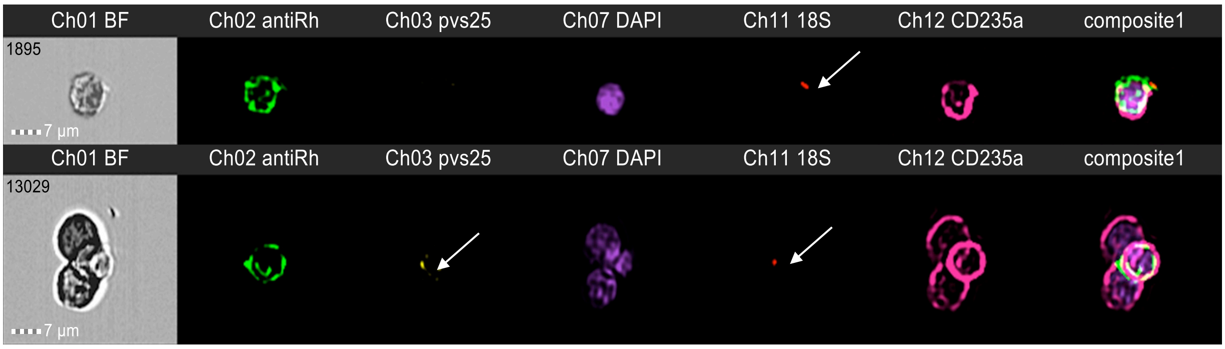

b

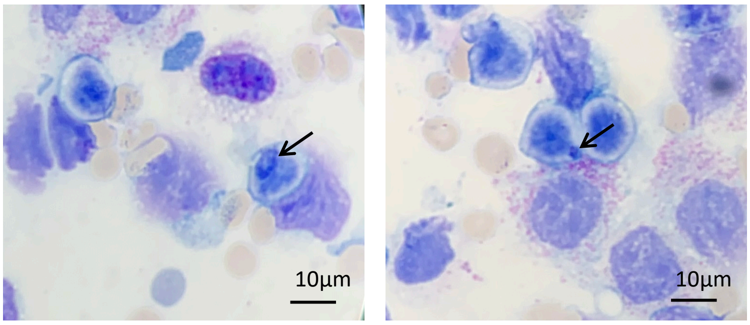

Supplementary Fig. 2 | HIS-HEry support sexual stages differentiation of *P vivax.*

a. FlowFISH imageStream profiles show (*top row*) asexual and (*bottom row*) sexual stages in endogenous human RBCs (RhD^{+}) from a day-7-infected HIS-HEry mouse. Cells were labeled for the RhD antigen (anti-Rh, FITC Chanel 02), gametocyte differentiation (Pvs25 RNA, Quasar 570 – Cy3, Chanel 03), nuclei (DAPI, Chanel 07), *P. vivax* 18S RNA (Pv18S, Quasar 670 – Cy5, Chanel 11), and the human erythrocyte marker (CD235a, APC-vio 770, Chanel 12). The composite image shows a merge of all the preceding images. These images are representative of *n* = 3 day-7-11 infected mice. **b**. Giemsa-stained thick smears of BM cells from a day-7-infected HIS-HEry mouse. Arrows indicate *P. vivax* parasites. Scale bar = 10 µm.

Supplementary Fig. 3

a

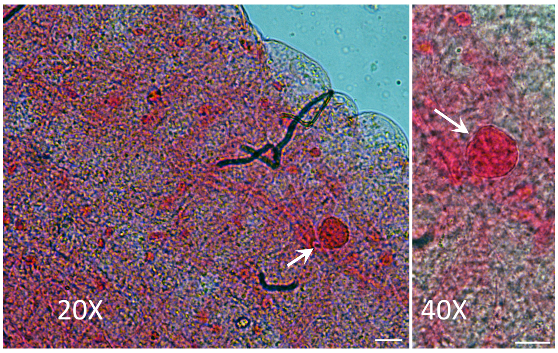

Mouse	dissected mosquitoes #	Total oocysts #	positive mosquitoes %
m1	28	2	7.14
m2	31	4	12.9

b

Pv isolate	Individual mouse (*n*=15)	# CytB RNA copies/ ml blood	# sporo from pooled mosquitoes	Mean sporo/ mosquito
**Pv1**	1	22	602	**12.04**
	2	17	1526	**30.52**
	3	75	1052	**21.04**
**Pv2+Pv3**	4	17918	346	**6.92**
**Pv3**	5	11	253	**8.43**
	6	54021	2349	**78.30**
	7	16500	147	**4.90**
	8	4591	896	**29.87**
**Pv4**	9	3	217	**3.62**
	10	5	3000	**50.00**
	11	6	1612	**26.87**
	12	4	0	**0.00**
**Pv5**	13	415	761	**15.22**
	14	5902	507	**10.14**
	15	194	588	**11.76**

Supplementary Fig. 3 | *P. vivax* gametocytes were transmitted to *Anopheles* mosquitos that fed on HIS-HEry mice and underwent development within the mosquitoes. **a-** (*Upper panel*) Microscopic image (20× magnification) shows mercurochrome staining of the midgut of a mosquito after feeding on a *P. vivax*-infected HIS-HEry chimera. Arrows show an oocyst. *Right panel* shows a magnification of the oocyst (40×). Scale bars = 30µm. These images are representative of oocysts found in mosquitoes fed on *n* = 2 different day-7-infected HIS-HEry mice (*Lower panel*). Data on oocyte detection in mosquitos for each of these 2 mice are shown. Values are the numbers of mosquitoes dissected for each mouse; the total oocysts obtained for each pool of mosquitoes that fed on that mouse; and the % of mosquitoes with oocysts. **b-** Characteristics of the 15 *P. vivax*-infected HIS-HEry mice (numbered 1 to 15) that were bitten by mosquitos; Pv: *P. vivax*; sporo: sporozoites. Source data are provided as a Source Data file.

Supplemental Figure 4

a

Gating strategy for phenotypic analysis of human erythroid cells:

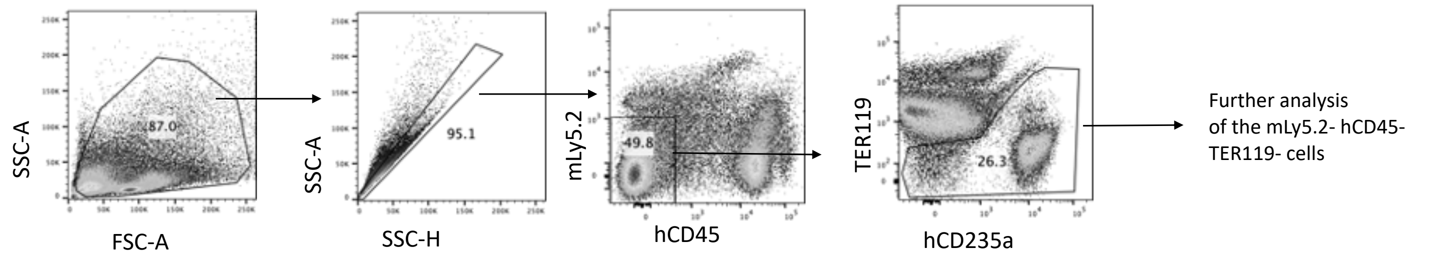

b Gating strategy for FlowFISH analysis of human erythroid cells:

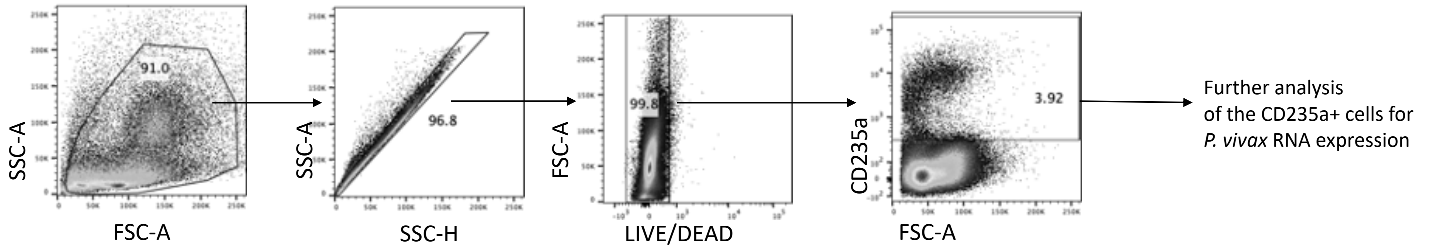

Supplementary Fig. 4 | FACS gating strategies. a- Representative gating for phenotypic analysis of human RBCs. **b-** Representative gating for FlowFISH analysis of human RBCs.

Supplementary Table 2 | Antibodies

Specificity	conjugate	clone	dilution	Source	catalog number
Anti-human Band3	PE	BRIC6	1/100	obtained from Bristol Institute for Transfusion Sciences	-
Anti-human CD235a	APC	HIR2	1/500	BD Biosciences	#551336
Anti-human CD235a	APC-Vio770	REA175	1/50	Miltenyi Biotec	#130-120-611
Anti-human CD235a	Pacific Blue	HI264	1/50	Biolegend	#349107
Anti-human CD235a	PercP-Cy5.5	HI264	1/50	Biolegend	#349109
Anti-human CD36	Vio Blue	REA760	1/50	Miltenyi Biotec	#130-110-745
Anti-human CD44	FITC	REA690	1/50	Miltenyi Biotec	#130-113-341
Anti-human CD45	AF700	HI30	1/100	Biolegend	#304023
Anti-human CD45	PE-CF594	HI30	1/100	BD Biosciences	#562312
Anti-human CD49d	APC	9F10	1/100	BD Biosciences	#555751
Anti-human CD49d	APC	9F11	1/50	Biolegend	#304307
Anti-human CD71	AF700	CY1G4	1/50	Biolegend	#334129
Anti-human CD71	FITC	AC102	1/50	Miltenyi Biotec	#130-098-781
Anti-human DARC (CD234)	PE-Vio770	REA376	1/50	Miltenyi Biotec	#130-105-685
Anti-human HbB	Alexa Fluor®647	37-8	1/50	Santa Cruz Biotechnology	#SC21757
Anti-human HbF	FITC	REA533	1/50	Miltenyi Biotec	#130-108-241
Anti-human IgG	DyLight 488	-	1/100	Invitrogen	#SA5-10126
Anti-human Rhesus D	purified	clone F5	1/40	provided by prof Yves Colin, INSERM U76 - INTS France	-
Anti-mouse Ly5.2	V500	104	1/100	BD Biosciences	#562130
Anti-mouse TER119	PE	REA847	1/50	Miltenyi Biotec	#130-112-723
Anti-mouse TER119	PercP	TER119	1/100	Biolegend	#116225
Anti-PvCSP	purified	MRA-184	1/500	hybridoma 2F2 obtained from BEI Resources, NIAID, NIH	-
Goat anti-mouse IgG	DyLight 488	-	1/2000	Invitrogen	#35502
FcR Blocking Reagent human	purified	-	1/50	Miltenyi Biotec	#130-059-901
Rat Anti-Mouse CD16/CD32 (Mouse BD Fc Block™)	purified	-	1/50	BD Biosciences	#553142

Supplementary Table 3 | RNA FISH Stellaris probes

Pvs25 RNA FISH probes	
**Probe #**	**Nucleotide sequence**
**1**	CAAGCTGTGGTAGGTATTCA
**2**	GTAAGCACGATGGCCAGAAG
**3**	CTTTGCGAAGGTGTGCTTAA
**4**	CATTTTTGCATTGGGTCTCC
**5**	GATTGCTCATTTGGACTACA
**6**	GTCGTTGCATTTGCATTCAA
**7**	TCGCAAGTGTTTTCATTTGC
**8**	GATTTGTGCAATCGCGTTTT
**9**	GTAGTCTCCACAGTTTTTAT
**10**	ATTCTGGTGTTTGCACACAC
**11**	GTAATGCTCTTTCCTCATTA
**12**	TACGGTGTACCCTAATATGC
**13**	ATGGAGTACACACCTCATTC
**14**	CACAGAACGCCGTTACATTT
**15**	TAAGATGCACTTTCCCTTTC
**16**	TGCTGTTCACATTAGCGGGA
**17**	CCTATATTACAAGAGCACAT
**18**	TTTAGATTCATCCAATGTGC
**19**	ATTCAGTTTTTCCTGGCTTT
**20**	TTTGCCTTACACTTCAACGT
**21**	TCTGAGTCTCTTTACATTCT
**22**	CTTCGCAACGCACTTGTAAT
**23**	CATGAGACTGTAAGCTGCTC
**24**	GTAGTATGCTGATTACTGCA
**25**	TGACATCATGAAGAAGGCGA
