# Supplementary material for: Humanized mice for investigating sustained Plasmodium vivax blood-stage infections and transmission
Source: Nat Commun. 2022 Jul 15;13:4123. doi: 10.1038/s41467-022-31864-6 (PMC9287384; doi:10.1038/s41467-022-31864-6)
Supplement: Supplementary file 2 — Reporting Summary [file 41467_2022_31864_MOESM2_ESM.pdf]

nature portfolio

Corresponding author(s): Sylvie GARCIA

Last updated by author(s): Jun 28, 2022

Reporting Summary

Nature Portfolio wishes to improve the reproducibility of the work that we publish. This form provides structure for consistency and transparency in reporting. For further information on Nature Portfolio policies, see our Editorial Policies and the Editorial Policy Checklist.

Statistics

For all statistical analyses, confirm that the following items are present in the figure legend, table legend, main text, or Methods section.

n/a	Confirmed	
☐	☒	The exact sample size (n) for each experimental group/condition, given as a discrete number and unit of measurement
☐	☒	A statement on whether measurements were taken from distinct samples or whether the same sample was measured repeatedly
☐	☒	The statistical test(s) used AND whether they are one- or two-sided<br>*Only common tests should be described solely by name; describe more complex techniques in the Methods section.*
☒	☐	A description of all covariates tested
☒	☐	A description of any assumptions or corrections, such as tests of normality and adjustment for multiple comparisons
☐	☒	A full description of the statistical parameters including central tendency (e.g. means) or other basic estimates (e.g. regression coefficient) AND variation (e.g. standard deviation) or associated estimates of uncertainty (e.g. confidence intervals)
☐	☒	For null hypothesis testing, the test statistic (e.g. F, t, r) with confidence intervals, effect sizes, degrees of freedom and P value noted<br>*Give P values as exact values whenever suitable.*
☒	☐	For Bayesian analysis, information on the choice of priors and Markov chain Monte Carlo settings
☒	☐	For hierarchical and complex designs, identification of the appropriate level for tests and full reporting of outcomes
☒	☐	Estimates of effect sizes (e.g. Cohen's d, Pearson's r), indicating how they were calculated

Our web collection on statistics for biologists contains articles on many of the points above.

Software and code

Policy information about availability of computer code

Data collection	Data of flow cytometric assays were collected by FACSDiva (version version 8.0.1) software. Data of Flow Imaging bassays were collected by Luminex corp Software Inspire version Mark II.
Data analysis	Statistical anaysis were performed using GraphPad Prism (version 9, GraphPad software) for the mean calculation and paired t-test and P value determination (Fig 3b). Data of flow cytometric assays were analyzed by Software FlowJo version 10.6.1. Data of Flow Imaging assays were analyzed by Luminex corp Software IDEAS version 6.2. Data of qRT-PCR were processed by Bio-Rad CFX Maestro software Version 4.1.2434.232.

For manuscripts utilizing custom algorithms or software that are central to the research but not yet described in published literature, software must be made available to editors and reviewers. We strongly encourage code deposition in a community repository (e.g. GitHub). See the Nature Portfolio guidelines for submitting code & software for further information.

Data

Policy information about availability of data

All manuscripts must include a data availability statement. This statement should provide the following information, where applicable:

- Accession codes, unique identifiers, or web links for publicly available datasets
- A description of any restrictions on data availability
- For clinical datasets or third party data, please ensure that the statement adheres to our policy

Data supporting the results of this manuscript are available within the article and the Supplementary Information file. The datasets generated and analyzed during this work are available from the corresponding author upon reasonable request. Source data are provided with this paper.

Field-specific reporting

Please select the one below that is the best fit for your research. If you are not sure, read the appropriate sections before making your selection.

☒ Life sciences ☐ Behavioural & social sciences ☐ Ecological, evolutionary & environmental sciences

For a reference copy of the document with all sections, see nature.com/documents/nr-reporting-summary-flat.pdf

Life sciences study design

All studies must disclose on these points even when the disclosure is negative.

Sample size	For in vivo infection studies (kinetics, de novo RBC infection), no statistical methods were used to predetermined the experimental sample size but rather it was determined on mice availability. We only kept mice for which the % of human CD45+ cells in the peripheral blood was ≥ 20 to ensure that mice were correctly engrafted during transplantation procedure. Each experiment was performed on a group of mice age-matched reconstituted at 1-4 day-old with 10 000-30 000 CD34+ cells from the same CB donor. Accordingly, sample size was based on the number of mice reconstituted with the same CB cells and thus depended on the number of CD34+ cells.<br>For BM transfer experiments (one donor into one recipient), the aim was to show that parasite could be transferred in variable settings.<br>For mosquito feeding, available infected mice were used.<br>For microscopic images (oocysts, GIEMSA staining), the aim was to qualitatively show parasites both in vertebrate and invertebrate hosts without any quantification purpose.<br>For in vitro qRT-PCR experiments, samples were analyzed as triplicates.
Data exclusions	No data has been excluded.
Replication	For all Figures, all experiments were performed several times independently as indicated in the figure legends, and all attempts to replicate the observations reported in the manuscript were successful. The number of biological replicates varied according to the experiments and are mentioned in the Figure legends.
Randomization	For each reconstitution experiment, all pups from the same litters were engrafted with human CD34+ cells from the same CB. Once adults (12-16 week-old), both male and female mice from the same litters were used and when required, distributed randomly in different experimental groups (for ex. different times post-infection), both male and females were used randomly provided they were reconstituted with the same CD34+ cells, provided that they presented a PB % human CD45+ cells ≥ 20.
Blinding	In vivo studies were performed in a blinded manner as infected versus non-infected mice were treated (housing, feeding, injection of clodronate liposomes) and analyzed similarly. Data were collected objectively, and analyzed using software with objective standards.

Reporting for specific materials, systems and methods

We require information from authors about some types of materials, experimental systems and methods used in many studies. Here, indicate whether each material, system or method listed is relevant to your study. If you are not sure if a list item applies to your research, read the appropriate section before selecting a response.

Materials & experimental systems

n/a	Involved in the study	
☐	☒	Antibodies
☒	☐	Eukaryotic cell lines
☒	☐	Palaeontology and archaeology
☐	☒	Animals and other organisms
☐	☒	Human research participants
☐	☒	Clinical data
☒	☐	Dual use research of concern

Methods

n/a	Involved in the study	
☒	☐	ChIP-seq
☐	☒	Flow cytometry
☒	☐	MRI-based neuroimaging

Antibodies

Antibodies used	Anti-human Band3 PE clone:BRIC6 obtained from Bristol Institute for Transfusion Sciences 1/100<br>Anti-human CD235a APC clone:HIR2 BD Biosciences cat#551336 1/500<br>Anti-human CD235a APC-Vio770 clone:REA175 Miltenyi Biotec cat#130-120-611 1/50<br>Anti-human CD235a Pacific Blue clone:HI264 Biolegend cat#349107 1/50<br>Anti-human CD235a PercP-Cy5.5 clone:HI264 Biolegend cat#349109 1/50<br>Anti-human CD36 Vio Blue clone:REA760 Miltenyi Biotec cat#130-110-745 1/50<br>Anti-human CD44 FITC clone:REA690 Miltenyi Biotec cat#130-113-341 1/50<br>Anti-human CD45 AF700 clone: HI30 Biolegend cat#304023 1/100<br>Anti-human CD45 PE-CF594 clone:HI30 BD Biosciences cat#562312 1/100

	Anti-human CD49d APC clone:9F10 BD Biosciences cat#555751 1/100<br>Anti-human CD49d APC clone:9F10 Biolegend cat#304307 1/50<br>Anti-human CD71 AF700 clone:CY1G4 Biolegend cat#334129 1/50<br>Anti-human CD71 FITC clone: AC102 Miltenyi Biotec cat#130-098-781 1/50<br>Anti-human DARC (CD234) PE-Vio770 clone:REA376 Miltenyi Biotec cat#130-105-685 1/50<br>Anti-human HbB Alexa Fluor®647 clone:37-8 Santa Cruz Biotechnology cat#SC21757 1/50<br>Anti-human HbF FITC clone:REA533 Miltenyi Biotec cat#130-108-241 1/50<br>Anti-human IgG DyLight 488 Invitrogen cat#SA5-10126 1/100<br>Anti-human Rhesus clone F5 provided by prof Yves Colin, INSERM U76 - INTS France 1/40<br>Anti-mouse Ly5.2 V500 clone:104 BD Biosciences cat#562130 1/100<br>Anti-mouse TER119 PE clone:REA847 Myltenyi cat#130-112-723 1/50<br>Anti-mouse TER119 PercP clone:TER119 Biolegend cat#116225 1/100<br>Anti-PvCSP FITC clone:MRA-184 (hybridoma 2F2 obtained from BEI Resources, NIAID, NIH) 1/500<br>Goat anti-mouse IgG DyLight 488Invitrogen cat#35502 1/2000<br>FcR Blocking Reagent human Miltenyi Biotec #130-059-901 1/50<br>Rat Anti-Mouse CD16/CD32 (Mouse BD Fc Block) BD Biosciences #553142 1/50
Validation	All antibodies are commercially available and validated by manufacturer (Biolegend, eBioscience); the dilutions used were recommanded by manufacturers and can be found on the websites of the manufacturers. The other non commercial anti-Band3, anti-RhD and anti-PvCSP antibodies were validated by Bristol Institute for Transfusion Sciences or Prof. Yves Colin, INSERM U76 - INTS France through publications and the dilution was communicated by the providers.

Animals and other organisms

Policy information about studies involving animals; ARRIVE guidelines recommended for reporting animal research

Laboratory animals	All mice were on the C57Bl/6 background. Rag2tm1Fwa IL2rgtm1Cgn B2mtm1Unc H2-Ab1tm1Doi Tg(HLA-DRA*0101,HLA-DRB1*0101)1Dma Tg(HLA-A2) Tg(SIRPA) Hc0 cKitW41d/d (CH1-2hSaW41) were obtained from the crossing of C57BL/6 cKitW41d/d mice with Rag2tm1Fwa IL2rgtm1Cgn B2mtm1Unc H2-Ab1tm1Doi Tg(HLA-DRA*0101,HLA-DRB1*0101)1Dma Tg(HLA-A2) Tg(SIRPA) Hc0 previously described by the authors (Malika Serra-Hassoun et al, JI, 2014). C57BL/6 CKitW41 d/d (B6KitW41) were kindly provided by C. Waskow, University of Dresden, Germany.<br>All mice were transplantation between 1-4 days after birth and both male and female mice were used for experiments at 12 - 16 week-old.<br>Mice were housed in filter top bonneted cages in isolator for breeding and reconstitution with human CD34+ cells, and housed in individually ventilated (IVC) for P. vivax conditions in 2 different A3 rooms with controlled access. Animals are provided food and water ad libitum.<br>Female Anopheles stephensi were either commercially provided by Radboud University Medical Center (RUMC), Nijmegen, The Netherlands, or they were reared in the Center for Production and Infection of Anopheles (CEPIA) at the Pasteur Institute, with standard procedures (SDA500 strain). Four-7 days Anopheles were used for blood feeding on infected-mice.
Wild animals	This study did not involve wild animals
Field-collected samples	This study did not involve samples collected from the field
Ethics oversight	Procedures involving mice were previously approved by local Animal Ethics Committees (CETEA # 089 at Institut Pasteur) and registered with the French authorities # #15341-2018053115015969.

Note that full information on the approval of the study protocol must also be provided in the manuscript.

Human research participants

Policy information about studies involving human research participants

Population characteristics	For non commercial CD34+ cells, cord bloods from de-identified from healthy donors (AP-HP, Hôpital Saint-Louis, Unité de Thérapie Cellulaire, CRB-Banque de Sang de Cordon, Paris, France – authorization number: AC-2016-2759) were used in some cases to process CD34+ cells.<br>Seven clinical P. vivax samples collected in northwestern Brazil in the context of an ongoing cohort study (ClinicalTrials.gov, NCT03689036) were leukocyte-depleted and cryopreserved in liquid nitrogen.<br>All donors were anonymized.
Recruitment	All participants were volunteers with no direct benefits from participation in this study.
Ethics oversight	For the CD34+ cord blood donors obtained with informed consent, the authorization number was AC-2016-2759.<br>Study protocols have been approved by the Institutional Review Board of the Institute of Biomedical Sciences, University of São Paulo, and by the National Human Research Ethics Committee of the Ministry of Health of Brazil (CAAE: 64767416.6.0000.5467); all patients provided written informed consent.

Note that full information on the approval of the study protocol must also be provided in the manuscript.

Clinical data

Policy information about clinical studies
All manuscripts should comply with the ICMJEguidelines for publication of clinical research and a completedCONSORT checklist must be included with all submissions.

Clinical trial registration	NCT03689036
Study protocol	available at clinicaltrials.gov with # NCT03689036
Data collection	Seven clinical P. vivax samples (de-identified and re-named Pv1-Pv5.3 throughout the manuscript) were collected in northwestern Brazil from patients tested positive for P. vivax infection. The parasitemias are provided in supplemental Table.
Outcomes	The present work used clinical samples collected and banked during the course of clinical trial # NCT03689036, but does not report the outcomes of the clinical trial. Consequently, the CONSORT checklist does not apply.

Flow Cytometry

Plots

Confirm that:

- [x] The axis labels state the marker and fluorochrome used (e.g. CD4-FITC).
- [x] The axis scales are clearly visible. Include numbers along axes only for bottom left plot of group (a 'group' is an analysis of identical markers).
- [x] All plots are contour plots with outliers or pseudocolor plots.
- [x] A numerical value for number of cells or percentage (with statistics) is provided.

Methodology

Sample preparation	BM and blood were collected on euthanized mice at d1, 7, 14 or 21 after infection. For BM, after dissection of the 2 femurs, tibias, and humerus, the bones were crushed, and cell suspensions were filtered through 40 uM filters before processing for Facs analysis. Because of infectious status, all samples from infection experiments were fixed in 2-4% PFA depending on the staining, before being collected by Flowcytometer or AMNIS.
Instrument	BD LSRFortessa™, Imaging cytometer MARKII (Luminex)
Software	Samples were collected on BD LSRFortessa™ using the Software DIVA version 8.0.1<br>Collected data were analyzed by FlowJo version 10.6.1<br>Alternatively, samples were collected on Imaging cytometer MARKII (Luminex) analyzer using the Luminex corp Software Inspire version Mark II.<br>Collected data were analyzed by Luminex corp Software IDEAS version 6.2.
Cell population abundance	When CB were processed in the laboratory using automacs positive selection, we obtained a purity of at least 95% of CD34+ determined by flow cytometry.
Gating strategy	Mononuclear live cells were gated according to FSC-A/SSC-A parameters, then according to SSC-A/SSC-W parameters for single cell analysis. When identicated, cells were stained with LIVE/DEAD™ Fixable Blue Dead Cell Stain Kit (Invitrogen) or 7AAD to exclude dead cells .<br><br>Then human red blood cells representation and development in the BM was stained using:<br>anti-human CD45 PE-CF594<br>anti-mouse Ly5.2 V500<br>anti-mouse TER119 percP<br>anti-human CD235a APC<br>anti-human CD44 FITC<br>anti-human CD71 AF700<br>anti-human CD36 Vio-Blue<br>anti-human DARC (CD234) PE-Vio770<br>CD45- Ly5.2- TER119- CD235a+ cells were analyzed for the expression of CD44, CD71, CD36, DARC<br><br>Alternatively, for BM and PB representation only we used:<br>anti-human CD235a APC<br>anti-human CD45 PE-CF594<br>anti-mouse TER119 PE<br>anti-mouse Ly5.2 V500<br>anti-human CD71 AF700<br><br>For human red blood cells maturation, BM cells were stained using:<br>1- anti-human CD235a Pacific Blue

anti-human CD49d APC
anti-human Band3 PE
7AAD

2- anti-human CD235a PercP-Cy5.5
anti-human HbF FITC
anti-huamn HbB AF647
7AAD

P. vivax RNA expression was analyzed upon FlowFISH processing using:
anti-CD235a
anti-Plasmodium 18S rRNA quasar-670
anti-Plasmodium Pvs25 mRNA quasar-570
anti-human RhD IgG / anti-IgG DyLight 488
Fixable Viability Dye LIVE/DEAD blue

☒ Tick this box to confirm that a figure exemplifying the gating strategy is provided in the Supplementary Information.
